# Vaccine- and Breakthrough Infection-Elicited Pre-Omicron Immunity More Effectively Neutralizes Omicron BA.1, BA.2, BA.4 and BA.5 Than Pre-Omicron Infection Alone

**DOI:** 10.3390/cimb45020112

**Published:** 2023-02-19

**Authors:** Eveline Santos da Silva, Jean-Yves Servais, Michel Kohnen, Victor Arendt, Georges Gilson, Therese Staub, Carole Seguin-Devaux, Danielle Perez-Bercoff

**Affiliations:** 1 HIV Clinical and Translational Research Unit, Department of Infection and Immunity, Luxembourg Institute of Health, 29 Rue Henri Koch, L-4354 Esch-sur-Alzette, Luxembourg; 2 Centre Hospitalier de Luxembourg, 4 Rue Ernest Barblé, L-1210 Luxembourg, Luxembourg

**Keywords:** SARS-CoV-2, neutralization, vaccination, hybrid immunity, breakthrough infection, Omicron BA.1 BA.2 BA.4 BA.5

## Abstract

Since the emergence of SARS-CoV-2 Omicron BA.1 and BA.2, several Omicron sublineages have emerged, supplanting their predecessors. Here we compared the neutralization of Omicron sublineages BA.1, BA.2, BA.4 and BA.5 by human sera collected from individuals who were infected with the ancestral B.1 (D614G) strain, who were vaccinated (3 doses) or with breakthrough infection with pre-Omicron strains (Gamma or Delta). All Omicron sublineages exhibited extensive escape from all sera when compared to the ancestral B.1 strain and to Delta, albeit to different levels depending on the origin of the sera. Convalescent sera were unable to neutralize BA.1, and partly neutralized BA.2, BA.4 and BA.5. Vaccinee sera partly neutralized BA.2, but BA.1, BA.4 and BA.5 evaded neutralizing antibodies (NAb). Some breakthrough infections (BTI) sera were non-neutralizing. Neutralizing BTI sera had similar neutralizing ability against all Omicron sublineages. Despite similar levels of anti-Spike and anti-Receptor Binding Domain (RBD) antibodies in all groups, BTI sera had the highest cross-neutralizing ability against all Omicron sublineages and convalescent sera were the least neutralizing. Antibody avidity inferred from the NT50:antibody titer ratio was highest in sera from BTI patients, underscoring qualitative differences in antibodies elicited by infection or vaccination. Together, these findings highlight the importance of vaccination to trigger highly cross-reactive antibodies that neutralize phylogenetically and antigenically distant strains, and suggest that immune imprinting by first generation vaccines may restrict, but not abolish, cross-neutralization.

## 1. Introduction

The Omicron lineage of SARS-CoV-2 comprises several sublineages. BA.1, BA.2 and BA.3 were first identifed in South Africa in November 2021. Between December 2021 and early January 2022, Omicron BA.1 rapidly outcompeted the Delta variant, which dominated the COVID-19 landscape across all continents at the time [1]. BA.1 was rapidly followed by the genetically distinct BA.2 sublineage, generating two overlapping peaks in most countries, including Luxembourg, in the winter and early spring 2022. BA.1 and BA.2 harbor 29 and 34 mutations, insertions and deletions in Spike, respectively, of which 21 are shared by the two sublineages. BA.3 shares mutations with both BA.1 and BA.2 but spread poorly compared to BA.1 and BA.2. Within a few months, other BA.2-derived sublineages, such as BA.4, BA.5 and BA.2.12.1 displaced BA.1 and BA.2. BA.4 and BA.5 have identical Spike sequences and differ by only three mutations in ORF7b, M (Membrane protein) and N (Nucleocapsid). Nevertheless, BA.5 has become the dominant variant in most countries. Since summer 2022, the Omicron landscape has expanded further. BA.2 sublineages BA.2.12.1, BA.2.75, BA.2.75.2, as well as BA.4, BA.5 and their offspring BA.4.6, BQ.1.1 and XBB.1 are gaining ground. These strains carry additional mutations which further increase their infectivity and antibody escape compared to the parental BA.2 [2,3,4,5,6,7,8,9,10].

While Omicron strains are less pathogenic and associated with lower fatality rates than Delta, the later strains BA.2.75 and BA.5 seem to gain pathogenicity compared to the early BA.1 and BA.2 [6,11,12,13,14,15,16,17,18,19,20,21,22]. The Omicron lineage typically features strongly enhanced transmissibility compared to Delta and pre-Omicron variants [3,4,5,6,23,24]. Increased transmissibility is due to its stronger docking to the receptor ACE2, and to its endocytosis-mediated, TMPRSS-2 independent entry into target cells, which also favors immune evasion [5,14,19,25,26,27,28,29,30,31,32,33], although BA.5 can also use the TMPRSS-2 route [24]. Furthermore, compensatory mutations have appeared in several strains to balance the replicative cost imposed by immune escape mutations (e.g., R493Q for F486V in BA.4/5 [2,34,35]). The Omicron Spike also adopts a distinct, more compact conformation and glycosylation patterns which shield it from type 1, 2 and 3 Neutralizing antibodies (NAbs) [33,36,37].

Typically, BA.1 and BA.2 show a dramatic drop in susceptibility to neutralization compared to the ancestral B.1 strain containing the Spike D614G mutation [25,26,27,28,38,39,40,41,42,43,44,45,46,47,48]. Sera from vaccinees who have received 2 vaccine doses do not cross-neutralize Omicron sublineages BA.1 and BA.2 [4,25,26,27,28,34,37,41,42,46,47,49,50]. Vaccination-induced and infection-induced NAbs wane after a few months. Booster vaccination (3rd and 4th doses) effectively restore NAbs and cellular responses against Omicron variants and protect against severe COVID-19 and death [28,29,34,42,45,46,50,51,52,53,54,55,56,57,58,59,60,61,62,63], but their durability is short [19,37,62,63]. BA.2 offspring BA.2.12.1 and BA.2.75 are modestly more resistant to NAbs than BA.2, while BA.2.75.2, BA.4 and BA.5 are typically more resistant to vaccine-elicited antibodies than BA.2 due to mutations such as R346T, L542R and F486V/S [2,4,8,9,10,19,34,35,52,63,64,65,66]. BA.2.75, BA.4 and BA.5 also resist antibodies elicited by Omicron BA.1 and BA.2 infection [2,3,19,34,35,52,65,66,67,68].

The emergence of antigenically distinct variants with increased infectivity and ability to evade immune responses elicited by prior infection or vaccination shapes both the pandemic landscape and clinical burden [2,65,69]. With the exponential increase in breakthrough infections due to Omicron, numerous studies have investigated cross-protection between Omicron sublineages. Epidemiological studies suggest that breakthrough infection with BA.1 and BA.2 confers some degree of cross-protection against infection with other Omicron sublineages like BA.2-offspring [70,71]. This increased protection may reflect better cross-neutralization due to antigenically closer strains, or the shorter time elapsed since infection [23,66,71,72,73,74]. Aside from vaccine-induced immunity, the cross-neutralization by pre-Omicron-elicited antibodies has been far less studied [41,43,44,59,75]. Yet, it is acknowledged that the first encounter with an antigen molds the immune response and this ‘immune imprinting’, also known as ‘original antigenic sin’, may limit and compromise the subsequent immune response. Depending on the original and challenge antigens, immune imprinting can be helpful if reactivation of existing memory B-cells rapidly provides antibodies that at least partially neutralize the virus, while new, variant-adapted NAbs are generated. Immune imprinting can, however, obstruct the generation of new, better adapted antibodies, either by neutralizing the antigen which is supposed to boost immunity (thus decreasing the impact of booster doses), or by skewing immunity to continue to produce antibodies against the past virus, impairing the generation of antibodies better suited to neutralize the new variant [2,65,75,76]. Imprinting has been beneficial up to Delta, but the completely different Omicron lineages evade antibodies and T-cells elicited by prior immunogens [2,3,24,68,75,76,77]. Conversely, studies on the cross-protection elicited by Omicron BA.1 infection alone against pre-Omicron and other Omicron sublineages consistently document poor cross-neutralization, highlighting the strong immune imprinting by this sublineage [3,24,34,44,52,65,68,76,78,79]. Obstructive immune imprinting has been described for BA.1, BA.2 and BA.5 breakthrough infections after vaccination with first generation vaccines as well [2,21,65,76,77], highlighting the antigenic distance between BA.1 and the other Omicron sub-lineages. In contrast, BA.2-elicited immunity confers cross-protection against BA.5 [19,66,80] and BA.2 offspring [19,66,80], emphasizing the beneficial impact of the reactivation of NAbs targeting common epitopes. Importantly, however, mixed immunity resulting from Omicron BA.1- and BA-2-breakthrough infections after 2 or 3 first generation vaccine doses or from reinfection after a first infection with a pre-Omicron strain elicit superior NAb as well as T-cell responses against all Omicron sublineages and pre-Omicron VOCs and confer better protection against severe forms of COVID-19 compared to boosters based on the original Wuhan strain or pre-Omicron (Beta or Delta) [3,5,19,24,41,44,55,68,69,76,78,79,81,82]. Accordingly, second generation bivalent vaccines combining ancestral and Omicron sequences (generally BA.1 or BA.4/BA.5) have been approved by the EMA and FDA, in September 2022, as boosters after first generation vaccines based on the original Wuhan strain.

In this context, and as BA.2 and its sublineages BA.4 and BA.5 continue to evolve, it is essential to have a clear view on the cross-neutralization of immune responses induced by infection, by vaccination and by both (hybrid immunity). Most studies have investigated the neutralizing ability of vaccinee sera and Omicron BTI sera against Omicron, but there are few studies comparing the neutralization of the main Omicron sublineages by pre-Omicron unvaccinated convalescent, vaccinee, and BTI sera. In this study, we focused on pre-Omicron immunity as it has been less investigated than Omicron breakthrough infection-elicited cross-immunity, although first-generation vaccines are still the most widespread. We aimed to compare different immune sera from convalescent, vaccinated, and breakthrough infections side by side, to assess whether there were qualitative and quantitative differences in their ability to neutralize Omicron sublineages. To that end, we compared the ability of sera from 58 individuals infected with the ancestral B.1 strain before vaccination (convalescent sera), 14 triple-vaccinated individuals, and 16 breakthrough infection patients (BTI) infected with Delta (14 patients) or Gamma (2 patients) to neutralize the pre-Omicron strains, B.1 (D614G) and Delta, and the four main Omicron sublineages: BA.1, BA.2, BA.4 and BA.5. We show that convalescent sera had the lowest neutralizing ability and BTI sera had the highest neutralizing ability against all strains. Convalescent sera from patients with moderate disease had better neutralizing ability. Overall, BA.1 was the most resistant to neutralization by all sera, BA.2 was the most sensitive to all sera, and BA.4 and BA.5 had intermediate resistance levels. However, sera from convalescent, vaccinee and BTI patients showed specificities in their ability to neutralize BA.5, which escaped neutralization by vaccinees better than infection-induced and hybrid immunity. Antibody avidity estimated from the NT50:antibody titer was also highest in BTI, providing some insight into the better efficacy of hybrid immunity.

## 2. Materials and Methods

### 2.1. Patient/Donor Samples

This study included sera from 58 unvaccinated patients (hereafter ‘convalescent sera’) infected between March and July 2020, sera from 14 individuals who received a mRNA booster dose between October 2021 and January 2022, and sera from 16 vaccinated patients with breakthrough infection (2 Gamma and 14 Delta) (hereafter ‘BTI sera’) who were infected between July 15th and September 20th 2021. All infected patients had RT-PCR-confirmed SARS-CoV-2 infection. The study was approved by the LIH Institutional Review Board (study number 14718697-NeutraCoV) and was performed in accordance with the 2018 Helsinki Declaration. Anonymized patient left-over samples collected at the Centre Hospitalier de Luxembourg (CHL) were used for the set-up of serological and virological tests in agreement with GDPR guidelines. No clinical data was available for any of the donors. The only available data for the unvaccinated patients was disease severity recorded by the clinician. Disease severity stratification was as follows: *Mild/asymptomatic* patients (7 patients) presented flu-like symptoms or no symptoms; patients with *Moderate* disease (17 patients) had fever, flu-like symptoms, anosmia, fatigue, and/or gastro-intestinal disturbances, but did not require hospitalization or oxygen supplementation; patients with *severe* or *critical* disease (34 patients) were admitted to the hospital, and required oxygen supplementation and/or intensive care. Convalescent sera were collected during acute infection (median 16.7 days after symptom onset, interquartile range [IQR] = 13.53–19.87 days). BTI sera were collected at the time of diagnosis, but time since symptom onset was not known. For BTI cases, data on the lineage of the infecting strain and the time since the 2nd vaccine dose were provided by CHL. For vaccinees, only the date of booster dose was available. Median time elapsed since booster (3rd) dose was 4 months (IQR = 2.26–6 months).

### 2.2. Cells

Vero-E6 cells (a kind gift from Dr. Thorsten Wolff, Influenza und respiratorische Viren, Robert Koch Institute, Berlin, Germany) were maintained in DMEM supplemented with 10% Foetal Bovine Serum (FBS), 2 mM L-Glutamine, 50 µg/mL Penicillin and 50 µg/mL Streptomycin (all from Invitrogen, Merelbeke, Belgium). For infection experiments, 2% FBS was used (hereafter referred to as Viral Growth Medium, VGM). HEK293T cells were from ATCC and were maintained in DMEM supplemented with 10% FBS, 50 µg/mL Penicillin and 50 µg/mL Streptomycin. HEK293T-ACE2-TMPRSS2 cells (SL222 HEK293T, GeneCopeia via Labomics, Nivelles, Belgium). They were maintained in HEK medium containing Puromycin (1 µg/mL) and Hygromycin (100 µg/mL).

### 2.3. Serology

The MesoScale Diagnostics (MSD) V-Plex COVID-19 Coronavirus Panel 1 serology kit (K15362U) was used according to the manufacturer’s recommendations to determine the IgG profile of sera (MesoScale Diagnostics, Rockville, MD, USA). This multiplex assay includes SARS-CoV-2 antigens (N, S, RBD, NTD) as well as Spike proteins from other Coronaviruses (SARS-CoV, MERS-CoV, OC43, HKU1) and Influenza A Hemagglutinin H3.

### 2.4. Virus Isolation and Titration

SARS-CoV-2 strains D614G and VOCs (Gamma, Delta and Omicron) were isolated from anonymized left-over patient nasopharyngeal swabs (NPS) collected from patients at the CHL and the Laboratoire Nationale de Santé to set-up the virological assays. For isolation, 500 µL of residual swab preservation medium was added to Vero-E6 cells (1.2 × 10^6^ cells) in VGM and the cytopathic effect (CPE) was monitored visually daily. Viral supernatant was used to constitute a viral stock by infecting Vero-E6 cells in a second passage. The viral supernatant from passage 2 was centrifuged and stored at −80 °C until use. All experiments were performed with the same viral stock. Viral strains present in the original material (swabs) were identified through next-generation sequencing and the Spike was resequenced after the second passage to verify sequence conservation. We isolated representative strains for B.1 (D614G, pre-VOC), Gamma, Delta, and Omicron (sublineages BA.1, BA.2, BA.4 and BA.5).

The 50% Tissue Culture Infectious Dose (TCID50) was assessed by titrating viral strains on Vero-E6 cells in sextuplicate wells as previously described [48]. Briefly, 10^4^ cells/well were infected with 200 µL of serial 10-fold dilutions of isolated virus (starting at 1:100 in VGM) for 72 h at 37 °C with 5% CO_2_. Virus-induced CPE was measured using the tetrazolium salt WST-8, which is cleaved to a soluble strongly pigmented formazan product by metabolically active cells (CCK-8 kit, Tebu-Bio, Antwerp, Belgium). Optical density at 570 nm was then measured. Virus-exposed wells were compared to uninfected wells (100% survival). The threshold for infection was set at 75% cell survival (i.e., all virus-exposed wells with <75% viable cells were considered infected) based on preliminary comparative experiments with visually recorded CPE and crystal violet staining. The TCID50 was calculated according to the Reed and Muench method [83].

### 2.5. Live-Virus Neutralization Assay

The live-virus neutralization assay has been described previously [48]. Briefly, serial two-fold dilutions of heat-inactivated (30 min at 56 °C) patient serum were incubated 1 h with 100 TCID50 of virus in VGM. The mixture (200 µL/well) was then inoculated on Vero-E6 cells (10^4^ cells/well in a 96-well microtitre plate) and cells were cultured for another 72 h at 37 °C with 5% CO_2_. A positive control (no serum) and an uninfected control (no serum–no virus) were included in each plate to assess maximum infection (no serum) and minimum (no virus) values. All infections were performed in triplicate wells. Virus-induced CPE was measured using the tetrazolium salt WST-8 as above. Percent survival was calculated relative to uninfected cells. The half-maximal inhibitory concentration for serum (IC50) was determined by inferring the 4-parameter nonlinear regression curve fit (GraphPad Prism v5) with unconstrained top and bottom values. The IC50 was log-transformed into 50% neutralizing titer (NT50) using the formula NT50 = 10^−IC50^. Sera with no neutralizing activity at the highest dilution tested (1:40) were considered non-neutralizing. The neutralizing capacity of convalescent and vaccinee sera was measured against B.1 (D614G strain) and Omicron sublineages BA.1, BA.2, BA.4 and BA.5. For BTI sera, the neutralizing ability of sera was also assessed against the infecting variant (i.e., Gamma BTI were evaluated against Gamma and Delta BTI against Delta). To ensure equivalent infection levels, a ‘back-titration’ was performed in each experiment with each of the viral strains. Briefly, the viral dilution used to infect cells in the presence or absence of serum dilutions was titrated as above, in 10-fold dilutions in VGM, and the TCID50 was calculated using the Reed and Muench formula to verify that the virus inoculum was 100 TCID50.

### 2.6. Pseudotype Preparation

HIV-based pseudotypes were generated as in [84] from the Firefly Luciferase-tagged HIV-1ΔenvΔnef backbone [85] complemented in trans with B.1, Omicron BA.1 or Omicron BA.2 Spike expression vectors lacking the 19 C-terminal amino acids containing the ER-retention signal (InvivoGen plv-spike-v11 and plv-spike-v12). HEK293T cells (1.2 × 10^6^ cells) were transfected with 2.5 µg HIV-1ΔenvΔnef and 0.5 (BA.1 and BA.2) or 1 µg (B.1) Spike expression vectors using JetPRIME. After 16 h, the medium was replaced and supernatants were collected after 48 h, centrifuged for 5 min at 4 °C and immediately used for neutralization assays.

### 2.7. Pseudotype-Based Neutralization Assay

Serial three-fold serum dilutions starting at 1:40 were incubated 30 min at 4 °C with B.1, Omicron BA.1 or Omicron BA.2 Spike pseudotypes. The serum/pseudotype mixture was then added to HEK293T-ACE2-TMPRSS2 cells (5 × 10^4^ cells/well) in DMEM containing 10% FBS for 60 h. Cells were then lysed using the Promega lysis buffer (E1531 from Promega, Belgium) and a freeze-thaw cycle. Then, Firefly Luciferase activity was assessed using the Firefly Luciferase Assay, following the manufacturer’s recommendations (E4550 from Promega, Belgium). Percent infection was calculated relative to infected cells in the absence of test serum. The IC50 was determined by inferring the 4-parameter nonlinear regression curve fit with unconstrained top and bottom values using GraphPad Prism v5 and was log-transformed into 50% neutralizing titer (NT50) as above.

### 2.8. Statistical Analyses

Statistical analyses were performed using GraphPad Prism v5. The Shapiro-Wilk test was used to verify distribution and all datasets were non-normally distributed. Differences between groups were compared using a Wilcoxon signed ranked test for comparisons between two groups and a Kruskal–Wallis signed-rank test followed by a Dunn’s post-hoc test for comparisons of three or more groups. A matched comparison was not applied in this case because data for all samples were not always available due to serum availability. Correlation coefficients (r) were determined using Spearman’s rank correlation. *p*-values < 0.05 were considered significant.

## 3. Results

### 3.1. Early Pandemic Convalescent Sera Poorly Neutralize Omicron Strains

#### 3.1.1. Cross-Neutralization of Convalescent Sera

**Figure 1:**
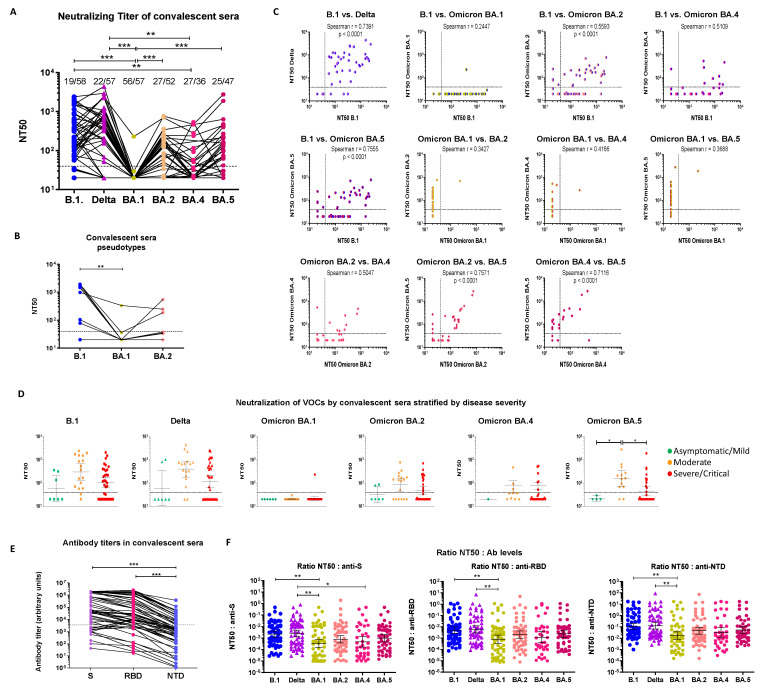
Neutralizing activity of pre-VOC unvaccinated SARS-CoV-2-infected convalescent sera against ancestral B.1, Delta and Omicron BA.1, BA.2, BA.4 and BA.5. (**A**) Comparison of NT50 between all strains. The infecting strain is indicated on the *x*-axis and with color codes: blue circles = B.1, purple triangles = Delta, gold hexagons = Omicron BA.1, pink hexagons = Omicron BA.2, orange hexagons = Omicron BA.4 and burgundy hexagons = Omicron BA.5. This color code is used throughout the figure and manuscript. The dotted line represents the 1:40 serum dilution cut-off. Sera which did not neutralize SARS-CoV-2 at the 1:40 dilution were considered non-neutralizing. The proportion of non-neutralizing sera is indicated above each data set. (**B**) NT50 for a subset of sera assessed using Luciferase-tagged HIV-1-Δ*env*Δ*nef*-Spike pseudotypes. All infections were performed in triplicate. (**C**) Correlation of NT50 between the tested strains. The Spearman correlation coefficient (r) is indicated in each panel. (**D**) NT50 of convalescent sera from patients with mild/asymptomatic (green), moderate (orange) or severe/critical (red) forms of COVID-19 against B.1, Delta, Omicron BA.1, BA.2, BA.4 and BA.5. (**E**) Anti-Spike, anti-RBD and anti-NTD antibody levels in convalescent sera. Antibody levels against Spike (purple), the Receptor Binding Domain (RBD) (pink) or the N-terminal domain (NTD) (blue) of Spike were measured in convalescent sera using the MSD V-plex platform for SARS-CoV-2. Antibody levels are reported as arbitrary units. (**F**) Ratios of NT50 to anti-S, (left panel), anti-RBD (middle panel) and anti-NTD (right panel) antibody levels. A Kruskal–Wallis test followed by a Dunn’s multiple comparison post-hoc test was used for comparisons between three or more groups (panels A-B, D-F). *p*-values < 0.05 were considered significant. *: *p* < 0.05; **: *p* < 0.01; ***: *p* < 0.001.

The convalescent sera used in this study were collected during the first SARS-CoV-2 wave from patients infected with the B.1 (Spike D614G) strain. All sera were collected during acute infection (median 16.7 days after symptom onset, interquartile range [IQR] = 13.5–19.9) and most were from patients with moderate or severe/critical COVID-19. Fifty percent neutralization titers (NT50) Geometric Mean Titer (GMT) were comparable between B.1 (GMT = 125.0, 95% Confidence interval (CI) [82.4, 189.7]) and Delta (GMT = 153.3, 95% CI [94.36, 249.0]) (Figure 1A and Supplementary Figure S1A). At the highest serum concentration tested (1:40), a similar proportion of sera (*p* > 0.05) failed to neutralize B.1 (19/58, 32.7%) and Delta (22/57, 38.6%) (Figure 1A). In contrast, all Omicron variants escaped neutralization by convalescent sera to some extent (Figure 1A and Supplementary Figure S2A–K). BA.1 was the most resistant to neutralization by convalescent sera (neutralizing GMT = 21.0 95% CI [19.3, 22.9]), with only one serum showing low-level neutralization, while all other sera were unable to even slightly neutralize Omicron BA.1 (Figure 1A and Supplementary Figure S1B). The NT50s of convalescent sera against BA.2 (GMT = 54.8, 95% CI [40.3, 74.5]), BA.4 (GMT = 37.2, 95% CI [26.6, 52.0]) and BA.5 (GMT = 60.4, 95% CI [40.8, 89.9]) were also lower than against B.1 (Figure 1A and Supplementary Figure S1C–E) and Delta (Figure 1A and Supplementary Figure S2B–D), but higher than BA.1 (Figure 1A and Supplementary Figure S1F–H). BA.2 and BA.5 had similar sensitivities to neutralization with over 50% of non-neutralizing sera (BA.2: 27/52, 52% and BA.5: 25/47, 53.2%). BA.4 was slightly although not significantly more resistant than BA.2 and BA.5 (*p* < 0.01 in both cases) and 75% (27/36) of the sera failed to neutralize this sublineage (Figure 1A, and Supplementary Figure S1B–K). The full escape from neutralization of BA.1 and the relative residual sensitivity of BA.2 to neutralization were confirmed using HIV-1-SARS-CoV-2 Spike pseudotypes on a subset of sera (Figure 1B).

It is noteworthy that there was no continuum in the cross-neutralizing ability of convalescent sera against different strains. For instance, some sera neutralized B.1 but failed to neutralize Delta (Supplementary Figure S1A) or Omicron BA.2, BA.4 or BA.5 (Supplementary Figure S1C–E). More interestingly, some sera neutralized BA.5 better than BA.2 (Supplementary Figure S1J). Most convalescent sera which cross-neutralized Omicron BA.2, BA.4, BA.5 had high NT50 (>350) against B.1 (Supplementary Figure S1C–E). Accordingly, there was a good, although imperfect, correlation between neutralizing activities of convalescent sera against B.1 and Delta (Spearman’s r = 0.7391, *p* < 0.0001) and a more modest correlation between B.1 or Delta and Omicron sublineages (Spearman r < 0.6) (Figure 1C and Supplementary Figure S2). Patients with moderate disease generally had higher neutralizing NT50 GMT than patients with mild or severe disease against all tested strains, although statistical support was reached only for BA.5 (Figure 1D).

#### 3.1.2. Serological Characterization of Convalescent Sera

To gain some qualitative insight on the antibodies mediating neutralization, we calculated the NT50:anti-S, NT50:anti-RBD and NT50:anti-NTD (N-terminal domain of S) ratios for all strains. This ratio subdivides the measured NT50 into the average neutralizing ability of individual antibodies and can thus be used as a surrogate to estimate antibody avidity [42,48]. As shown in Figure 1E, nearly all patients had detectable antibodies against S, the RBD and the NTD, although antibody levels varied substantially between patients. The NT50:anti-S, NT50:anti-RBD and N50:anti-NTD ratios were comparable for B.1 and Delta and were ~1 log_10_ lower for Omicron BA.1 (*p* < 0.01) (Figure 1F). The three ratios were also lower for BA.2, BA.4 and BA.5, in line with the corresponding neutralizing titers. This observation suggests that antibodies elicited by infection with early SARS-CoV-2 partially cross-react with Omicron BA.2 and its sublineages BA.4 and BA.5 better than with BA.1.

### 3.2. Sera from Boosted Vaccinees Retain Partial Neutralizing Activity against All Omicron Sublineages

#### 3.2.1. Cross-Neutralization of Triple-Vaccinated Sera

**Figure 2:**
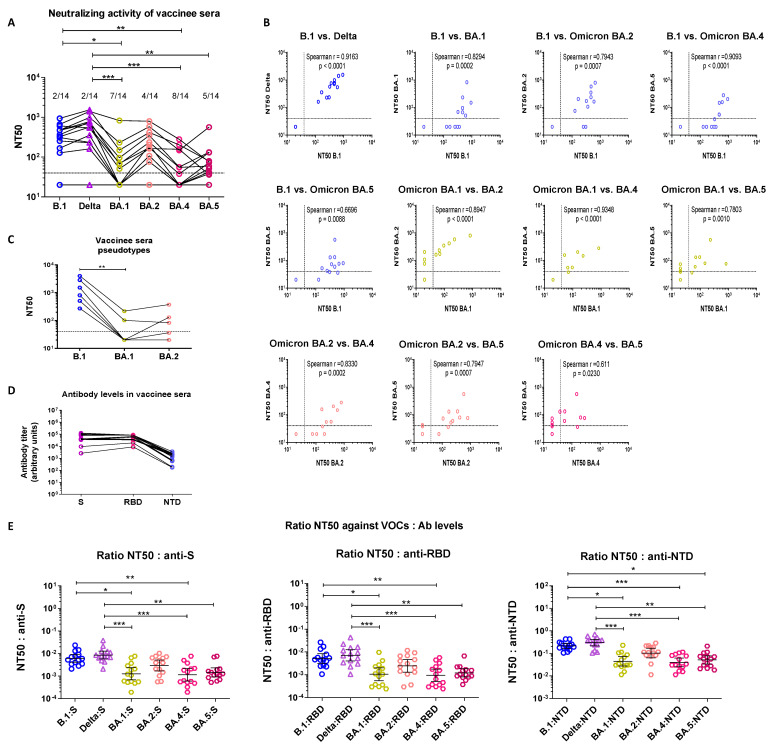
Neutralizing activity of sera from triple-vaccinated individuals against ancestral B.1, Delta and Omicron BA.1, BA.2, BA.4 and BA.5. (**A**) Comparison of NT50 between all strains. The infecting strain is indicated on the *x*-axis and with color codes: blue open circles = B.1, purple open triangles = Delta, gold open hexagons = Omicron BA.1, pink open hexagons = Omicron BA.2, orange open hexagons = Omicron BA.4 and burgundy open hexagons = Omicron BA.5. The dotted line represents the 1:40 serum dilution cut-off. Sera which did not neutralize SARS-CoV-2 at the 1:40 dilution were considered non-neutralizing. The proportion of non-neutralizing sera is indicated above each data set. (**B**) Correlation of NT50 between the tested strains. The Spearman correlation coefficient (r) is indicated in each panel. (**C**) NT50 for a subset of sera assessed using Luciferase-tagged HIV-1-Δ*env*Δ*nef*-Spike pseudotypes. All infections were performed in triplicate. (**D**) Anti-Spike, anti-RBD and anti-NTD antibody levels in vaccinee sera. Antibody levels against Spike (purple), the Receptor Binding Domain (RBD) (pink) or the N-terminal domain (NTD) (blue) of Spike were measured in vaccinee sera using the MSD V-plex platform for SARS-CoV-2. Antibody levels are reported as arbitrary units. (**E**) Ratios of NT50 to anti-S, (left panel), anti-RBD (middle panel) and anti-NTD (right panel) antibody levels. A Friedman test followed by a Dunn’s multiple comparison post-hoc test was used for comparisons between three or more groups (panels (**A**,**C**–**E**)). *p*-values < 0.05 were considered significant. *: *p* < 0.05; **: *p* < 0.01; ***: *p* < 0.001.

Next, we assessed the neutralizing ability of sera from 14 triple-vaccinated individuals against the same SARS-CoV-2 VOCs. The time elapsed between booster dose and sampling varied between 15 days and 6 months (median = 4 months, IQR = 2.26–6). Infection history is not known. The NT50 GMT for B.1 and Delta were 246.8, 95% CI (124.4, 489.8) and 344.6, 95% CI (155.6, 763.3), respectively. The correlation between NT50s of both pre-Omicron VOCs was very good (Spearman r = 0.9163, *p* < 0.0001), indicating that sera which neutralized B.1 also neutralized Delta (Figure 2A,B). As shown in Figure 2A and Supplementary Figure S3A, 2/14 vaccinee sera (14%) were unable to neutralize the ancestral B.1 and Delta. Time since vaccination for these two non-neutralizing samples was 5 and 6 months, reflecting the loss of neutralization activity over time. The NT50 GMTs were lowest for BA.1 (NT50 = 51.1 (95% CI [25.6, 100.5]), BA.4 (NT50 = 46.1, 95% CI [25.7, 82.7]) and BA.5 (NT50 = 58.9 (95% CI [34.9, 99.5]). The drop in NT50 compared to B.1 reached statistical significance for BA.1 and BA.4 and more than 50% of sera failed to neutralize these two Omicron sublineages at the highest dilution tested (7/14 for BA.1, 8/14 for BA.4 and 5/14 for BA.5) (Figure 2A). Despite lower NT50 GMT compared to B.1 (Figure 1A), BA.2 remained more sensitive to neutralization by vaccinee sera than the other Omicron sublineages (NT50 = 119.2 (95% CI [55.5, 255.8])), with only 2 non-neutralizing sera at the 1:40 dilution. In contrast to convalescent sera, the neutralizing ability of vaccinee sera against B.1 extended to other VOCs, i.e., sera which poorly neutralized B.1 generally failed to neutralize Omicron sublineages, and neutralizing sera retained some neutralizing ability against Omicron strains as well (Figure 2A). Accordingly, the side-by-side comparison of sera NT50 against pre-Omicron (B.1 and Delta) and Omicron VOCs showed overall excellent Spearman correlation coefficients (Spearman r > 0.66) (Figure 2B and Supplementary Figure S4A–D). Similar results for BA.1 and BA.2 were again recorded with pseudotypes (Figure 2C).

#### 3.2.2. Serological Characterization of Triple Vaccinee Sera

We then calculated the NT50:antibody ratios for vaccinees. All vaccinees had detectable antibodies against S, the RBD and the NTD in serum (Figure 2D). Overall, antibody levels spanned a narrower range than convalescent sera and most antibodies against S targeted the RBD. Again, the NT50:antibody level ratio was comparable for B.1 and Delta, but was markedly lower (~1 log_10_) for Omicron BA.1, BA.4 and BA.5 for all antibodies (anti-S, anti-RBD and anti-NTD) (Figure 2E), illustrating the lower affinity of vaccine-induced antibodies for these Omicron sublineages. Despite identical Spike sequences, the NT50:Ab ratio was lower for BA.4 than for BA.5, reflecting subtle differences in the susceptibility of these two sublineages to neutralization. The NT50:antibody ratios for Omicron BA.2 were intermediate, nicely recapitulating the NT50 profiles. These figures indicate that antibodies elicited by first generation vaccines retain sufficiently high affinity for BA.2 Spike determinants, whilst BA.1, BA.4 and BA.5 have evolved to further escape binding and thereby neutralization by pre-Omicron-elicited antibodies.

Taken together, these results document that antibodies elicited by first generation vaccines partially retain neutralizing ability against Omicron sublineages 4 months after the 3rd dose, despite a significant drop compared to the ancestral B.1. They also clearly show qualitative differences between infection-elicited and vaccine-elicited antibodies: vaccination remains more effective against BA.2 than against the other Omicron sublineages, while convalescent sera were unable to cross-neutralize BA.1 but similarly neutralized Omicron BA.2, BA.4 and BA.5 (compare Figure 1A and Figure 2A). They also suggest that vaccination induces more broadly reactive antibodies than acute infection.

### 3.3. Breakthrough Infection Sera Have Distinct Neutralization Profiles and Retain Cross-Neutralizing Ability against All Omicron Sublineages

#### 3.3.1. Cross-Neutralization of Pre-Omicron BTI Sera

**Figure 3:**
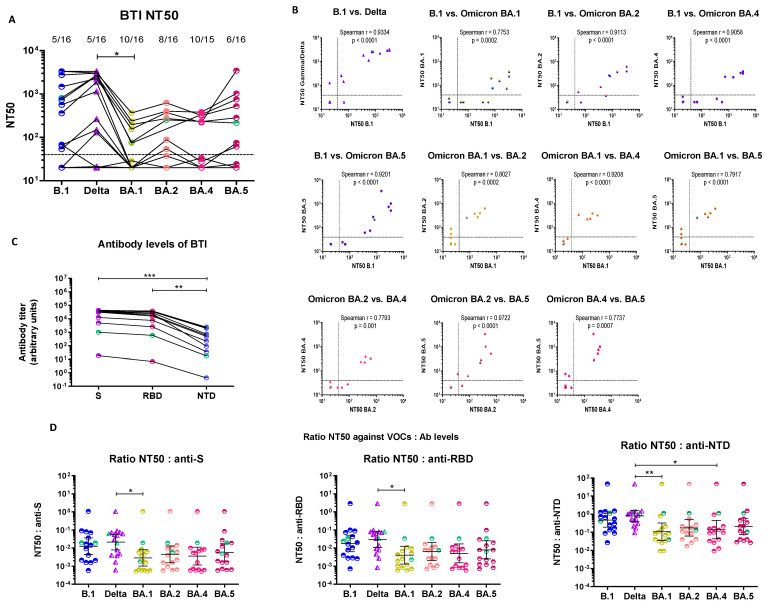
Neutralizing activity against ancestral B.1, Delta and Omicron BA.1, BA.2, BA.4 and BA.5 of sera from vaccinated individuals (2 doses) with Delta or Gamma breakthrough infection (BTI). (**A**) Comparison of NT50 between all strains. The infecting strain is indicated on the *x*-axis and with color codes: partially filled blue circles = B.1, partially filled purple triangles = Delta, partially filled gold hexagons = Omicron BA.1, partially filled pink hexagons = Omicron BA.2, partially filled orange hexagons = Omicron BA.4 and partially filled burgundy open hexagons = Omicron BA.5. Gamma-BTI are identified with green symbols in all panels. This color code is used throughout the figure. The dotted line represents the 1:40 serum dilution cut-off. Sera which did not neutralize SARS-CoV-2 at the 1:40 dilution were considered non-neutralizing. The proportion of non-neutralizing sera is indicated above each data set. (**B**) Correlation of NT50 between the tested strains. The Spearman correlation coefficient (r) is indicated in panel. (**C**) Anti-Spike, anti-RBD and anti-NTD antibody levels in BTI sera. Antibody levels against Spike (purple), the Receptor Binding Domain (RBD) (pink) or the N-terminal domain (NTD) (blue) of Spike were measured in vaccinee sera using the MSD V-plex platform for SARS-CoV-2. Antibody levels are reported as arbitrary units. (**D**) Ratios of NT50 to anti-S, (left panel), anti-RBD (middle panel) and anti-NTD (right panel) antibody levels. A Kruskal–Wallis test followed by a Dunn’s multiple comparison post-hoc test was used for comparisons between three or more groups (panels **A**,**C**,**D**). *p*-values < 0.05 were considered significant. *: *p* < 0.05; **: *p* < 0.01; ***: *p* < 0.001.

Hybrid immunity conferred by vaccination and infection together was reported to be superior to immunity elicited by infection or vaccination alone [3,24,40,41,67,68,72,76,78,79,86,87,88]. As such, we further investigated the susceptibility of the four Omicron sublineages to sera from 16 vaccinated individuals with breakthrough infection (BTI) with pre-Omicron VOCs (2 infected with Gamma and 14 with Delta). BTI patients were infected between 15 July and 20 September 2021, when Gamma and Delta were the main circulating VOCs in Luxembourg. The median time elapsed since the 2nd vaccine dose was 3.1 months [CI = 2.1–4.5]. Most sera were collected at the time of diagnosis but time since symptom onset is unknown. As shown in Figure 3A (and Supplementary Figure S5A), half the sera were strongly neutralizing against B.1 and the infecting VOC (Gamma or Delta), while the other half was fully non-neutralizing (5 sera: 4 Delta, 1 Gamma) or poorly neutralizing (3 sera). Overall, NT50s did not differ significantly between B.1 and the infecting VOC (Gamma for Gamma-BTI cases and Delta for Delta-BTI cases): GMT B.1 = 194.6, 95% CI [65.6, 577.0]) and GMT Gamma/Delta = 329.5, 95% CI [102.0, 1064.0], *p* > 0.05). All Omicron lineages escaped neutralization by BTI sera to some extent (Figure 3A and Supplementary Figure S5B–E), as follows: NT50 GMT: BA.1 NT50 = 44.4, 95% CI (25.0, 78.9), BA.2 NT50 = 70.7, 95% CI (32.3, 154.9), BA.4 NT50 = 51.6, 95% CI (25.4, 104.9), and BA.5 NT50 = 87.9, 95% CI (34.4, 224.6) (Supplementary Figures S5B–E and S6A–D). Similar to what we observed for convalescent and vaccinee sera, the drop in neutralization was more pronounced for BA.1, although this trend did not reach statistical significance, likely due to the low number of cases and the high proportion of non-neutralizing BTI sera (Figure 3A and Supplementary Figure S5F–H). Strikingly, Delta- and Gamma-BTI sera retained good neutralizing ability against BA.5, as illustrated by the excellent correlation score (Spearman r = 0.9201) (Figure 3B). BA.4 was slightly more resistant to neutralization than BA.2 and BA.5 (*p* < 0.05 in both cases) (Supplementary Figure S5I,K), as previously recorded for unvaccinated convalescent sera.

Like the cross-neutralizing profile of vaccinee sera, the cross-neutralizing profile of BTI sera was also relatively well maintained across strains. Sera which neutralized B.1 at dilutions higher than 1:80 had similar or higher neutralizing ability against the corresponding infecting VOC (Supplementary Figure S5A) and six retained some, although weaker, cross-neutralization against all Omicron sublineages. Conversely, most sera that did not neutralize B.1 also failed to neutralize the infecting VOC (1 Gamma-BTI and 3 of 4 non-neutralizing Delta-BTI sera) (Supplementary Figure S5A) and Omicron sublineages (Figure 3A and Supplementary Figures S5B–K and S6A–D). Accordingly, there was a very good correlation between the NT50s of BTI sera against B.1, the infecting VOC (Gamma or Delta) (Spearman r = 0.9334, *p* < 0001) and against the Omicron sublineages (Spearman r > 0.77 in all cases) (Figure 3B and Supplementary Figure S6A–D).

#### 3.3.2. Serological Characterization of Pre-Omicron BTI Sera

Despite distinct neutralization profiles, all but one Delta-BTI sera had antibodies against S, the RBD and the NTD (Figure 3C). The NT50:antibody ratios were again ~1 log_10_ higher for B.1 and Delta compared to Omicron strains, although statistical support was reached only between the infecting strain and BA.1 for the three ratios and for the NT50:anti-NTD ratio as well for BA.4 (Figure 3D).

Overall, Delta-BTI sera showed a distinct neutralization profile, with two groups of sera, neutralizing or non-neutralizing. A similar profile was observed for Gamma-BTI (1 neutralizing and 1 non-neutralizing). There was a good cross-neutralization across strains, including Omicron, as recorded for vaccinee sera and a small loss in antibody avidity estimated from the NT50:antibody level ratio.

### 3.4. Comparison of Convalescent, Vaccinee and BTI Sera

**Figure 4:**
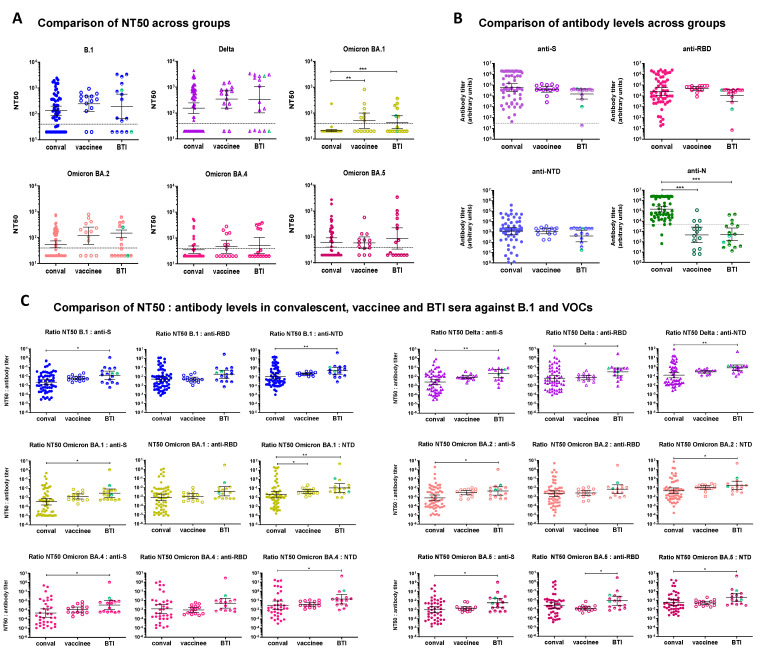
Comparison of neutralizing activities of convalescent, vaccinee and BTI sera against B.1, Delta and Omicron BA.1, BA.2, BA.4 and BA.5. (**A**) Comparison of NT50s from convalescent, vaccinee and BTI sera for B.1, Delta, Omicron BA.1, BA.2, BA.4 and BA.5. NT50 of convalescent, vaccinee and BTI sera were compared for each strain. The infecting strain is indicated above each panel. The dotted line represents the 1:40 serum dilution cut-off. Sera which did not neutralize SARS-CoV-2 at the 1:40 dilution were considered non-neutralizing. (**B**) Comparison of antibodies against the Nucleocapsid (N), Spike (S), RBD and NTD in convalescent, vaccinee and BTI sera. For BTI sera, the Gamma-BTI are represented with black circles and the Delta-BTI are represented in purple for anti-S antibodies, pink for anti-RBD antibodies, blue for anti-NTD antibodies and green for anti-N antibodies. Anti-N and anti-S antibodies were also estimated with the Euroimmun assay for BTI and only 3 BTI sera had anti-N antibodies. The grey dotted lines in the panels for anti-S and anti-N antibody levels mark the threshold between anti-S and anti-N-positive and negative sera based on the Euroimmun assay. (**C**) Comparison of the NT50:antibody level ratios for convalescent, vaccinee and BTI sera. The NT50:anti-S, NT50:anti-RBD and NT50:anti-NTD ratios for convalescent, vaccinee and BTI sera against each strain are compared. The infecting strain is indicated above each group of panels. For BTI sera, the Gamma-BTI are represented with green symbols. For all analyses, differences between groups were compared using a Kruskal–Wallis test followed by a Dunn’s multiple comparison post-hoc test. *p*-values < 0.05 were considered significant. *: *p* < 0.05; **: *p* < 0.01; ***: *p* < 0.001.

Because there were shared trends and differences between the three groups of sera included in this study, we set out to compare the NT50s and NT50:antibody ratios from convalescent, vaccinee and BTI sera. Overall, convalescent sera had the lowest NT50 GMT and BTI sera had the highest NT50 GMT (Figure 4A). This trend held true for all strains, although it was more pronounced for BA.1 and BA.2 and statistical support was reached only for Omicron BA.1, reflecting the fact that BA.1 fully escaped neutralization by convalescent sera at the highest serum concentration tested (1:40 dilution). Vaccinee and BTI sera had similar NT50 for most strains. Of note, neutralization of BA.4 and BA.5 by convalescent, vaccinee and BTI sera was comparable, reflecting the better cross-neutralization of BA.5 by convalescent sera (Figure 1A), and the poor neutralizing ability of vaccinee sera against BA.4 (Figure 2A). It is worth mentioning that convalescent sera were collected at the time of acute infection, while 4 months had elapsed since the last vaccine dose for vaccinees. BTI sera were collected at the time of diagnosis, which corresponds to 3 months after vaccination. Therefore, neutralizing ability 4 months after the 3rd vaccine dose remained higher than that conferred by early infection against most strains tested (B.1, Delta, Omicron BA.1 and BA.2) (Figure 4A).

Although convalescent sera had lower NT50s, the levels of antibodies targeting Spike and the NTD were comparable between convalescent and vaccinee sera (Figure 4B). BTI sera tended to have overall lower antibody levels compared to convalescent and vaccinee sera (Figure 4B). Anti-RBD antibodies tended to be higher in vaccinee sera compared to convalescent and BTI sera (Figure 4B), likely reflecting the open conformation of the Spike in mRNA vaccines. Given the time elapsed since the third vaccine dose, these figures are likely underestimates. Therefore, similar antibody levels ensured higher neutralization in vaccinated individuals (both uninfected and BTI) than in acutely infected unvaccinated patients. Although the time since symptom onset is not known, the levels of anti-N antibodies (Figure 4B) confirm that, in most cases, infection of BTI was recent and had likely triggered a rapid boost of anti-S, anti-RBD and anti-NTD antibodies from memory B-cells, but not yet caused the appearance of anti-N antibodies.

The NT50:anti-S and NT50:anti-NTD ratios in BTI sera were higher than those in convalescent and vaccinee sera (Figure 4C). The difference was statistically significant between BTI and convalescent sera for all strains. Again, these differences are likely underestimated, given that half the BTI have no neutralizing ability. The NT50:anti-S and NT50:anti-NTD ratios of vaccinee sera were intermediate between convalescent sera and BTI (Figure 4C). Anti-RBD antibodies showed a similar trend between convalescent and BTI sera, but statistical support was reached only for Gamma/Delta, i.e., for the infecting strain. Importantly, the NT50:RBD ratio for vaccinee sera was similar to convalescent sera, indicating that the slightly higher anti-RBD levels likely account for the higher NT50, suggesting a more targeted immune response.

Together, these results indicate that breakthrough infection elicits neutralizing antibody responses with higher avidity than both infection alone and booster vaccination, including against Omicron sublineages. The higher cross-neutralizing ability of BTI sera is likely due to the superior avidity of antibodies (Figure 4C). This may explain, at least partly, the superiority of hybrid immunity over infection or vaccination alone and is consistent with ongoing affinity maturation after infection and vaccination [40,42,43,86,88,89,90].

## 4. Discussion

In this study, we compared the neutralizing ability of pre-Omicron immune sera from convalescent, triple-vaccinated and pre-Omicron breakthrough infection individuals against four Omicron sublineages. All sera exhibited a marked or full drop in neutralizing ability against all Omicron sublineages compared to B.1 and Delta (Figure 4). BA.1 was the most resistant to neutralization and BA.2 the least (Figure 1A, Figure 2A and Figure 3A). BA.4 and BA.5 had intermediate resistance levels overall, albeit with differences between the two sublineages and between the groups of sera (Figure 4). Convalescent and BTI sera poorly neutralized BA.1, neutralized BA.4 slightly better and similarly neutralized BA.2 and BA.5 (BA.1 < BA.4 < BA5~BA.2). Vaccinee sera, in contrast, neutralized Omicron BA.5 significantly less efficiently than BA.2 (Supplementary Figure S3J) (BA.1 < BA.4 < BA.5 < BA.2). This is in line with prior studies reporting the higher escape of BA.5 from NAbs [2,4,19,34,35,52,64,65,66,91]. The high evasion capacity of Omicron BA.1, BA.4 and BA.5 to immunity from vaccination with two, three or four doses is extensively documented [2,3,4,19,21,24,28,34,42,45,46,49,50,51,52,53,54,55,56,57,59,61,62,63,64,65,66,67,68,75]. However, our results reveal that convalescent sera retain some neutralizing ability against the BA.5 sublineage, in line with a prior report [92]. Despite this, convalescent sera were unable to neutralize BA.1, in agreement with previous reports [44,59,78] and poorly neutralized BA.4. The inability of convalescent sera to neutralize BA.1 somewhat mirrors the restricted cross-neutralizing ability of BA.1-elicited antibodies against pre-Omicron and other Omicron lineages [3,24,34,44,52,65,68,76,78,79,80,93], and highlights that BA.1 is antigenically very distant from all other VOCs and triggers strongly imprinted immune responses. Accordingly, one study found that the BA.1 booster triggers the activation of naïve B-cells rather than memory B-cells [77], while others record a small proportion of naïve B-cell activation [2,65,69,90]. This observation also suggests that different antigens (infection or mRNA vaccines) lead to slightly different immune imprints, confirming a recent study [92].

Surprisingly, BA.4 and BA.5 had different NAb escape profiles as well. BA.4 and BA.5 share identical Spike sequences and differ by 3 mutations located in ORF7b (L11F in BA.4), in N (P151S in BA.4) and in the Membrane protein M (D3S in BA.5). The widespread use of pseudotypes does not allow the distinction of differences between BA.4 and BA.5 and only few reports have investigated the susceptibility of BA.4 to NAbs using live virus [3,68]. These mutational differences confer BA.5 a growth advantage over BA.4. This is consistent with the observation that BA.4 has not spread much in Luxembourg, and that BA.5 has become the dominant variant in Luxembourg and elsewhere, indicating that infectivity and transmissibility, rather than NAb escape, were the main selective drivers, as previously reported [4,24]. However, our findings indicate that these mutations, located outside of Spike, may also allow a better escape from NAbs through indirect mechanisms which would warrant further exploration.

Despite differences between the neutralizing abilities of convalescent, BTI and vaccinee sera, all groups of sera showed some resilience towards BA.2 (Figure 1, Figure 2 and Figure 3). The literature on the susceptibility of BA.2 to neutralization is still controversial, as some studies record similar or higher resistance to neutralization for BA.2 compared to BA.1 [4,19,50,52,63,64,78] while others record a less dramatic drop in neutralization for BA.2 compared to BA.1 [4,24,37,45,66,76]. These differences probably ensue from different vaccination/infection histories and from different experimental and calculation approaches (target cells, live virus versus pseudotypes, PRNT50/PRNT90, NT50). Our findings with both live virus and pseudotypes agree with the latter. Furthermore, the NT50:antibody ratios in vaccinee sera were higher for BA.2 compared to the other Omicron sublineages, indicative of residual affinity (Figure 2E). The fact that BA.2 remains partially susceptible to neutralization by convalescent and vaccinee sera while BA.4 and BA.5 more efficiently eluded humoral responses suggests that its selective advantage lied in increased transmissibility at a time where vaccine and infection coverage was still moderate. As increasing numbers of individuals acquired immunity through infection or vaccination, the trade-off between infectivity and immune escape must have shifted. Accordingly, several BA.2-derived offspring are spurring in a BA.5-dominated context. The acquisition of supplementary mutations in Spike, such as R346T (BA.2.75.2, BQ.1.1 and XBB.1) or F486P (BA.2.10.4 and BA.4.6) increases their ability to evade NAb and illustrates the strength of the immune selective pressure imposed on the virus [4,8,9,10,34,52,66]. While some of these mutations arise independently in different sublineages (converging evolution), others appear to be more specific [2]. The increasing number of mutations accumulated by these variants underscores not only their role in incrementing antibody evasion and viral fitness, but also the plasticity of the SARS-CoV-2 genome. More importantly, they clearly indicate that SARS-CoV-2 has not reached an evolutionary threshold and that there is still room for evolution.

NAb levels are thought to be predictive of protection [94,95] and are inversely correlated with viral load [78]. In our study, convalescent sera and vaccinee sera harbored comparable anti-S, anti-RBD and anti-NTD antibody levels, while BTI tended to have slightly lower antibody levels (Figure 4B). These quantitative similarities likely partly ensue from sampling times (acute infection for convalescent, ~4 months after 3rd vaccine dose for vaccinees and at diagnosis for BTI which corresponds to ~3 months after 2nd vaccine dose). However, they also reveal profound qualitative differences in antibodies from unvaccinated convalescent sera compared to vaccinee sera, in agreement with other recent studies, albeit based on different BTI infections [3,43,44,68]. First, sera from vaccinated individuals (both uninfected and BTI) which neutralized B.1 also cross-neutralized Delta (Figure 2A and Figure 3A and Supplementary Figures S3A and S5A) and partially neutralized Omicron sublineages, including the notoriously resistant BA.1, BA.4 and BA.5 (Figure 2 and Figure 3). Convalescent sera, in contrast, had a less straightforward cross-neutralization pattern, as some non-neutralizing sera against B.1 were able to neutralize Delta or Omicron sublineages (Figure 1A and Supplementary Figure S1A–K). Second, antibodies in convalescent sera mediated lower neutralization against all strains than vaccinees and BTIs, indicative of superior efficacy and avidity of vaccination (with or without breakthrough infection) over infection alone in agreement with previous studies by us and by others [3,48,59,63,68,78,87]. This was particularly striking for Omicron BA.1, which all but one convalescent serum were unable to neutralize (Figure 1A). In all groups of donors, the NT50:anti-RBD ratio of vaccinee sera was lower for BA.1, BA.4 and BA.5 than for pre-Omicron strains and BA.2 (Figure 4C), reflecting a much lower avidity of antibodies targeting the RBD against these sublineages, as previously reported by others [3,24,80]. Antibody avidity (estimated from the NT50:antibody ratios) was slightly (although not significantly) lower in convalescent than in vaccinee sera, and was significantly higher in BTI sera (Figure 4C). This observation likely reflects the fact that convalescent sera were collected during acute infection, while there had been time for affinity maturation in vaccinee and BTI sera. It is noteworthy that the NT50:anti-RBD ratio was significantly higher in BTI than in convalescent sera only for Delta. This observation can reflect affinity maturation after vaccination and/or after infection or beneficial immune imprinting which results in very effective binding to the Delta RBD, although it is not possible to distinguish between the two processes from our data. Together, our findings not only confirm the superiority of hybrid immunity [3,24,40,41,52,67,68,72,76,78,79,86,87,88], including that conferred by pre-Omicron antigens towards Omicron strains, in agreement with previous studies [3,43,44,48,59,63,68,78,79,87], but also highlight distinctive qualitative features and cross-neutralizing profiles between antibodies elicited by infection and by vaccination. These differences may be because mRNA vaccines expose Spike in an open conformation and thus favor antibodies targeting the RBD, while infection triggers a broader spectrum of antibodies. They may also ensue from affinity maturation, which occurred in vaccinated and BTI individuals but not in acutely infected patients.

The time since vaccination/infection and the infecting variant dictate the level and quality of immune responses to mRNA booster vaccination [72,82]. Ongoing affinity maturation shapes immune responses elicited by infection [86,96,97,98,99] and by vaccine boosters, including those based on the original Wuhan strain [40,42,43,86,88,89,90]. Accordingly, the time elapsed between vaccination and breakthrough infection is proportional to the breadth of the resulting antibody response [74,88]. We do not have information on the time window between the second and the third vaccine doses in vaccinees included in this study but, based on the typical vaccination protocols deployed in Luxembourg and in Europe at the time, it is most likely that it exceeded 6 months, thus longer than the 4 months that elapsed between the boost and breakthrough infection in the BTI group. However, we found no correlation between the time window between vaccination (3rd dose) and breakthrough infection and NT50 against the infecting strain (not shown), but this may be due to the low number of BTI cases.

It is worth mentioning that as of February 2023, more than half (58%) of the population in Luxembourg has received three doses of vaccine administrated during the winter 2021 but only 12.3% has received the 2nd booster (ECDC COVID-19 Vaccine Tracker https://qap.ecdc.europa.eu/public/extensions/COVID-19/vaccine-tracker.html#uptake-tab accessed on 14 February 2023). Emerging strains must possess a selective advantage over the strains dominating the landscape they emerge in. Therefore, in a context of suboptimal or waning immunity, viral fitness and transmissibility will most likely be the main driving forces. Variants with higher replicative capacity will readily outgrow existing variants, as was the case for BA.2 in early 2022. As immune pressure increases due to vaccine campaigns or to epidemic bursts, immune evasion becomes the driving force, and variants cumulating resistance mutations will most likely emerge. Presently, mutations that contribute to both immune escape and transmissibility emerge in the BA.2 and BA.5 backgrounds, make the epidemic landscape more complex. A possible threat is the emergence of new variants from a totally distinct lineage, in response to the strong immune pressure imposed by the high number of infections with BA.2-derived lineages.

The data reported here only includes pre-Omicron sera and thus sheds light on pre-Omicron cross-immunity, which has been much less studied than cross-immunity between Omicron infections. It would have been insightful to compare the cross-neutralization and antibody avidity in sera from pre-Omicron and Omicron breakthrough infections, in order to better assess if and to what extent Delta and Omicron breakthrough infections mold immune responses. One shortcoming of this study is that it only includes a small number of vaccinee and BTI cases infected with pre-Omicron VOCs. This is due to the relatively low occurrence of breakthrough infections before the Omicron burst. There is high vaccine coverage in Luxembourg and thus good protection against severe forms of COVID-19, resulting in fewer hospitalizations. Indeed, breakthrough infections and hospital admissions increased from Wuhan to Delta but decreased overall with Omicron, despite increased infectivity of the later Omicron sublineages [23,24]. Finally, another drawback of this study is the lack of sequential sampling. Indeed, sequential sampling could shed light on the NAb levels over time and the duration of the protection conferred by infection, vaccination and hybrid immunity.

## 5. Conclusions

In conclusion, vaccine-induced pre-Omicron immunity boosted by 3rd dose or by breakthrough infection remains at least partially effective against all tested Omicron lineages, albeit with a substantial decrease compared to the ancestral B.1. The level of neutralization 4 months after the 3rd vaccine dose is comparable to that induced by infection alone at the time of acute infection against B.1. Our findings also reveal that antibodies elicited by pre-Omicron infection alone less efficiently bind Spike determinants and, consequently, less effectively neutralize Omicron sublineages than those triggered by vaccination. Furthermore, these antibodies did not have a broad cross-neutralizing spectrum. They neutralized BA.2 and BA.5 better than the other Omicron lineages, while vaccine elicited antibodies had a more linear cross-neutralizing pattern, which was less effective against BA.5. This diversity is increasing further with the exponential increase of infections due to Omicron and immune pressure, adding more intricate layers to the immune landscape.

## Data Availability

Data available upon request.

## Supplementary Materials

The following supporting information can be downloaded at: https://www.mdpi.com/article/10.3390/cimb45020112/s1, Supplementary Figure S1: Pairwise comparison of 50% neutralizing titers (NT50) of convalescent sera against tested strains; Supplementary Figure S2: Pairwise comparison and correlation of 50% neutralizing titers (NT50) of convalescent sera against Delta and Omicron sublineages; Supplementary Figure S3: Pairwise comparison of 50% neutralizing titers (NT50) sera from triple-vaccinated individuals against tested strains; Supplementary Figure S4: Pairwise comparison of 50% neutralizing titers (NT50) of sera from triple-vaccinated individuals against Delta and Omicron sublineages; Supplementary Figure S5: Pairwise comparison of 50% neutralizing titers (NT50) of BTI sera against tested strains, Supplementary Figure S6: Pairwise comparison and correlation of 50% neutralizing titers (NT50) of BTI sera against Delta and Omicron sublineages.
