# Supplementary material for: Vaccine- and Breakthrough Infection-Elicited Pre-Omicron Immunity More Effectively Neutralizes Omicron BA.1, BA.2, BA.4 and BA.5 Than Pre-Omicron Infection Alone
Source: Curr Issues Mol Biol. 2023 Feb 19;45(2):1741–61. doi: 10.3390/cimb45020112 (PMC9955496; doi:10.3390/cimb45020112)
Supplement: Supplementary file 1 [file cimb-45-00112-s001.zip › cimb-2220056-supplementary.pdf]

current issues in molecular biology

MDPI

Supplementary materials

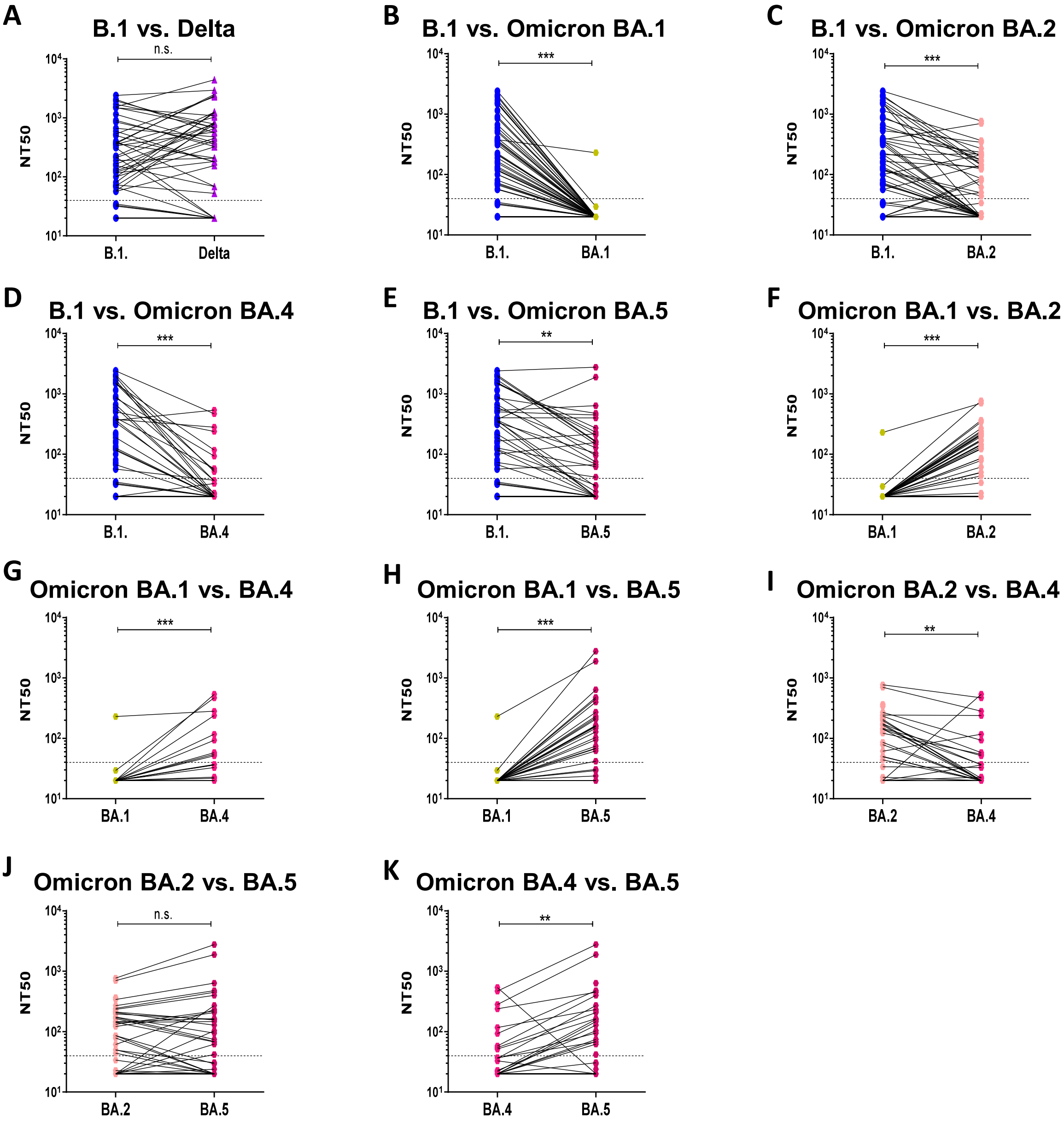

Supplementary Figure S1. Pairwise comparison of 50% neutralizing titers (NT50) of convalescent sera against tested strains. Comparison of NT50 B.1 versus Delta (**A**), B.1 versus Omicron BA.1 (**B**), B.1 versus Omicron BA.2 (**C**), B.1 versus Omicron BA.4 (**D**), B.1 versus Omicron BA.5 (**E**), Omicron BA.1 versus Omicron BA.2 (**F**), Omicron BA.1 versus Omicron BA.4 (**G**), Omicron BA.1 versus Omicron BA.5 (**H**), Omicron BA.2 versus Omicron BA.4 (**I**), Omicron BA.2 versus Omicron BA.5 (**J**) and Omicron BA.4 versus Omicron BA.5 (**K**). Cells were infected with the strain indicated on the x-axis. The dotted line represents the 1:40 serum dilution cut-off. Sera which did not neutralize SARS-CoV-2 at the 1:40 dilution were considered non-neutralizing. Differences between groups were compared using a Wilcoxon signed rank test. P-values < 0.05 were considered significant. *: $p<0.05$; **: $p<0.01$; ***: $p<0.001$.

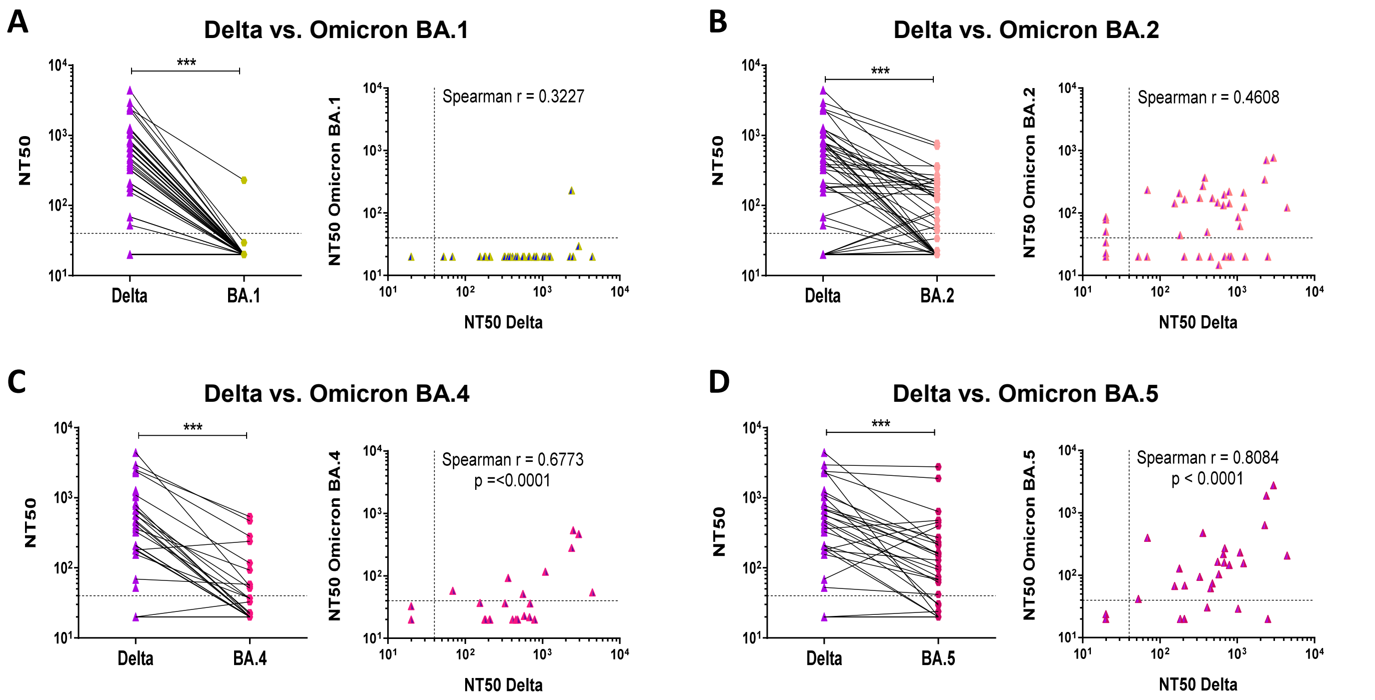

Supplementary Figure S2. Pairwise comparison and correlation of 50% neutralizing titers (NT50) of convalescent sera against Delta and Omicron sublineages. NT50 between Delta and Omicron sublineages BA.1 (**A**), BA.2 (**B**), BA.4 (**C**) and BA.5 (**D**) were compared. Cells were infected with the strain indicated on the x-axis. Paired samples (left panel),-and Spearman correlation (right panel) are shown. The dotted line represents the 1:40 serum dilution cut-off. Sera which did not neutralize SARS-CoV-2 at the 1:40 dilution were considered non-neutralizing. Differences between groups were compared using a Wilcoxon signed rank test. The Spearman correlation coefficient (r) is indicated. P-values < 0.05 were considered significant. *: p<0.05; **: p<0.01; ***: p<0.001.

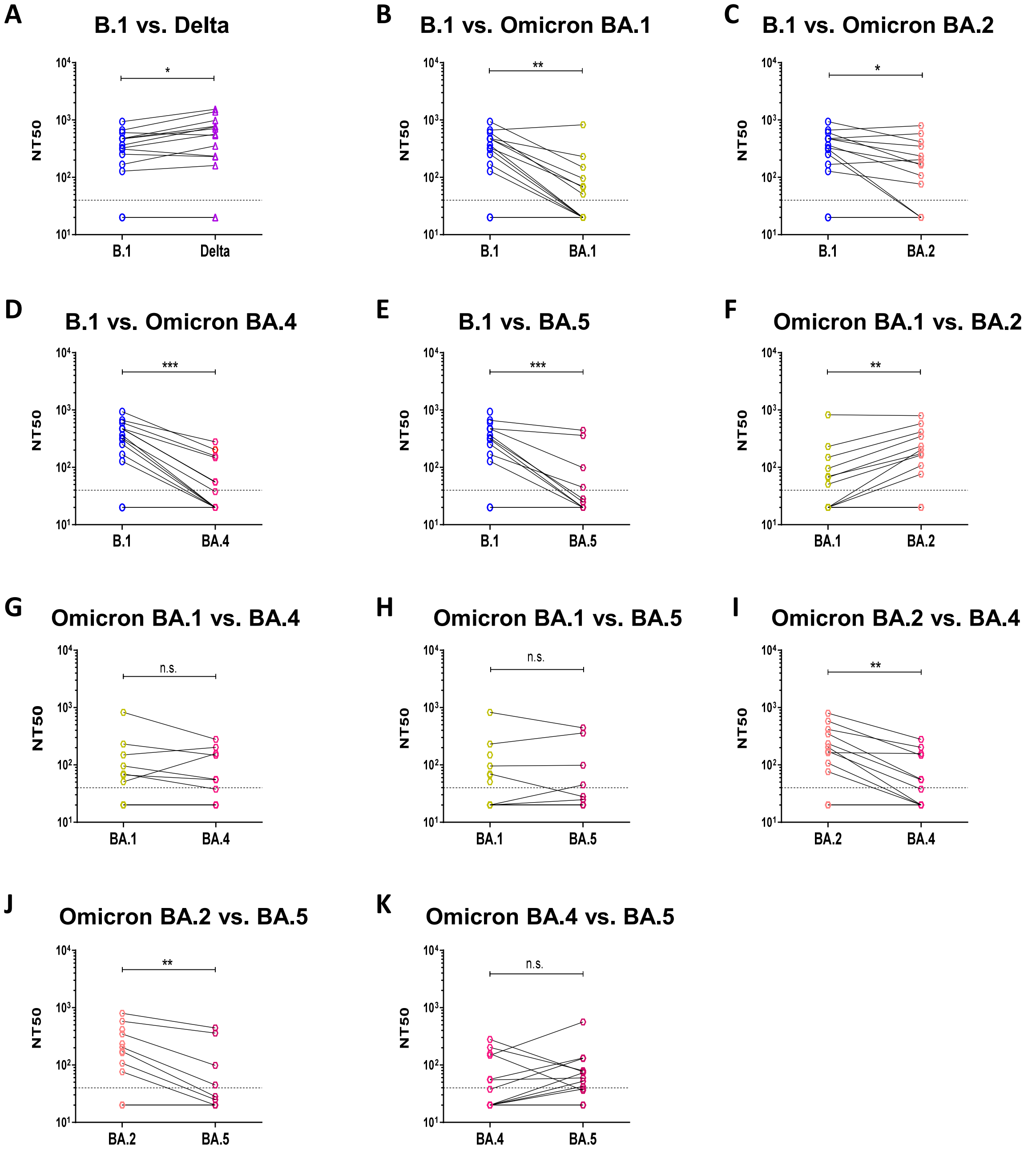

Supplementary Figure S3. Pairwise comparison of 50% neutralizing titers (NT50) sera from triple-vaccinated individuals against tested strains. Comparison of NT50 B.1 versus Delta (**A**), B.1 versus Omicron BA.1 (**B**), B.1 versus Omicron BA.2 (**C**), B.1 versus Omicron BA.4 (**D**), B.1 versus Omicron BA.5 (**E**), Omicron BA.1 versus Omicron BA.2 (**F**), Omicron BA.1 versus Omicron BA.4 (**G**), Omicron BA.1 versus Omicron BA.5 (**H**), Omicron BA.2 versus Omicron BA.4 (**I**), Omicron BA.2 versus Omicron BA.5 (**J**) and Omicron BA.4 versus Omicron BA.5 (**K**). Cells were infected with the strain indicated on the x-axis. The dotted line represents the 1:40 serum dilution cut-off. Sera which did not neutralize SARS-CoV-2 at the 1:40 dilution were considered non-neutralizing. Differences between groups were compared using a Wilcoxon signed rank test. P-values < 0.05 were considered significant. *: p<0.05; **: p<0.01; ***: p<0.001.

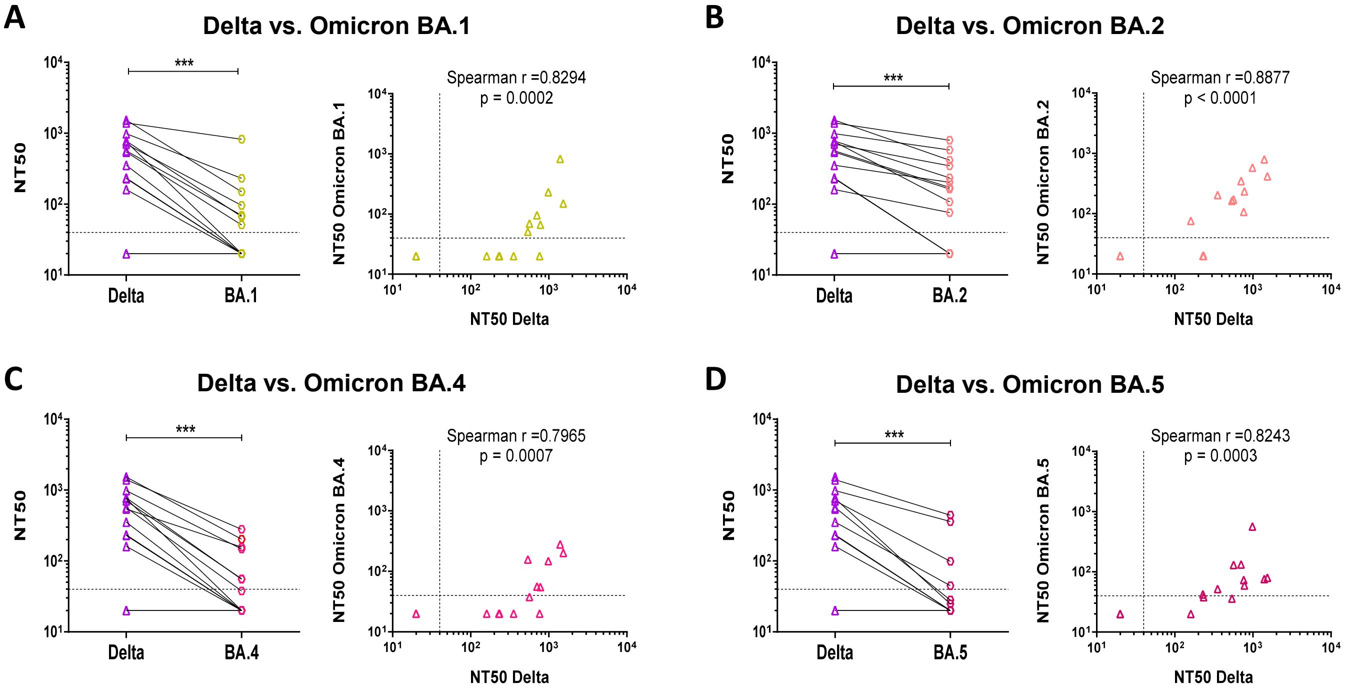

Supplementary Figure S4. Pairwise comparison of 50% neutralizing titers (NT50) of sera from triple-vaccinated individuals against Delta and Omicron sublineages. NT50 between Delta and Omicron sublineages BA.1 (**A**), BA.2 (**B**), BA.4 (**C**) and BA.5 (**D**) were compared. Cells were infected with the strain indicated on the x-axis. Paired samples (left panel) and Spearman correlation (right panel) are shown. The dotted line represents the 1:40 serum dilution cut-off. Sera which did not neutralize SARS-CoV-2 at the 1:40 dilution were considered non-neutralizing. Differences between groups were compared using Wilcoxon signed rank test. The Spearman correlation coefficient (r) is indicated. P-values < 0.05 were considered significant. *: $p<0.05$; **: $p<0.01$; ***: $p<0.001$.

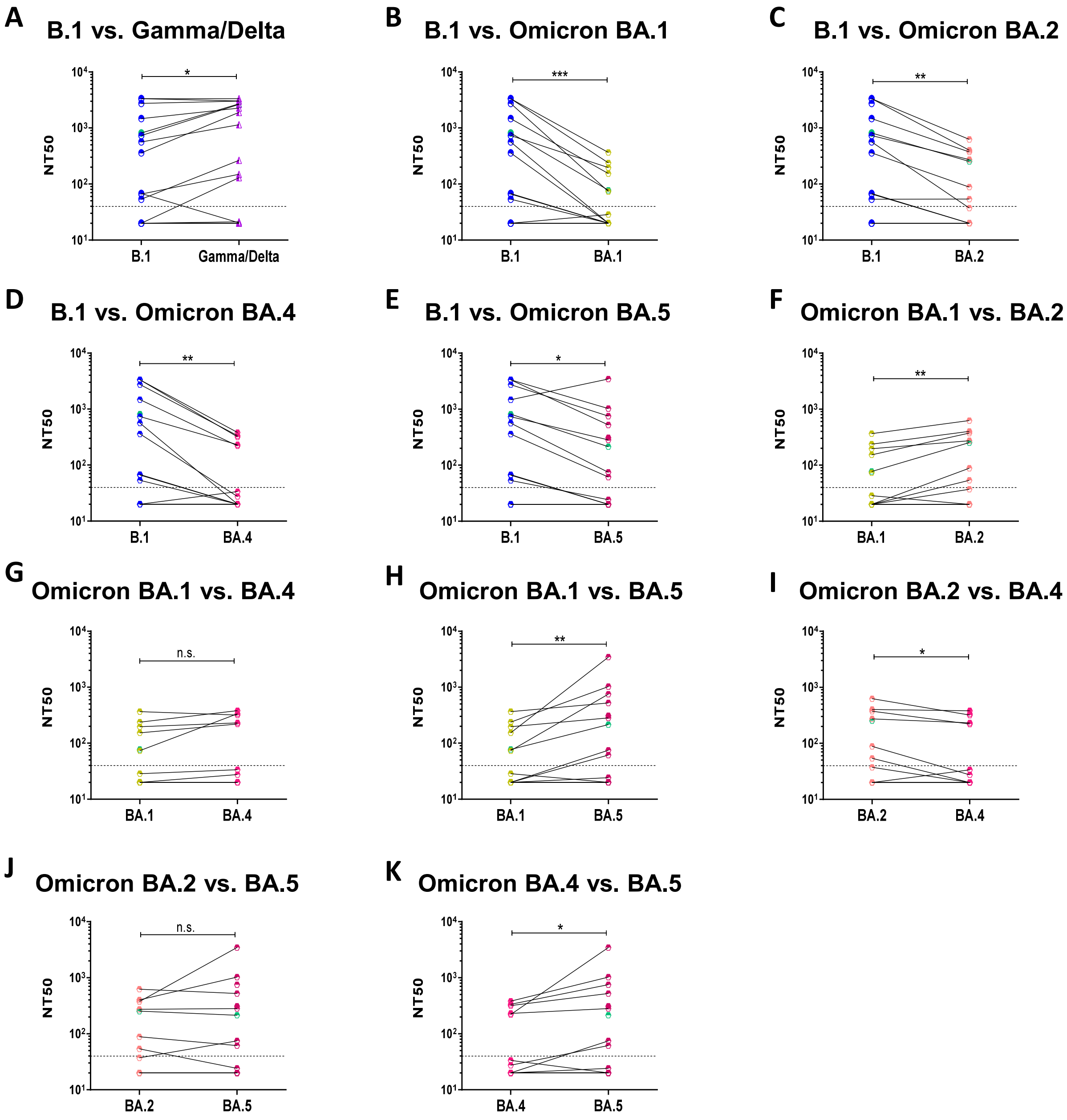

Supplementary Figure S5. Pairwise comparison of 50% neutralizing titers (NT50) of BTI sera against tested strains. Comparison of NT50 B.1 versus Gamma/Delta (**A**), B.1 versus Omicron BA.1 (**B**), B.1 versus Omicron BA.2 (**C**), B.1 versus Omicron BA.4 (**D**), B.1 versus Omicron BA.5 (**E**), Omicron BA.1 versus Omicron BA.2 (**F**), Omicron BA.1 versus Omicron BA.4 (**G**), Omicron BA.1 versus Omicron BA.5 (**H**), Omicron BA.2 versus Omicron BA.4 (**I**), Omicron BA.2 versus Omicron BA.5 (**J**) and Omicron BA.4 versus Omicron BA.5 (**K**). Cells were infected with the strain indicated on the x-axis. The dotted line represents the 1:40 serum dilution cut-off. Sera which did not neutralize SARS-CoV-2 at the 1:40 dilution were considered non-neutralizing. Differences between groups were compared using a Wilcoxon signed rank test. P-values < 0.05 were considered significant. *: p<0.05; **: p<0.01; ***: p<0.001.

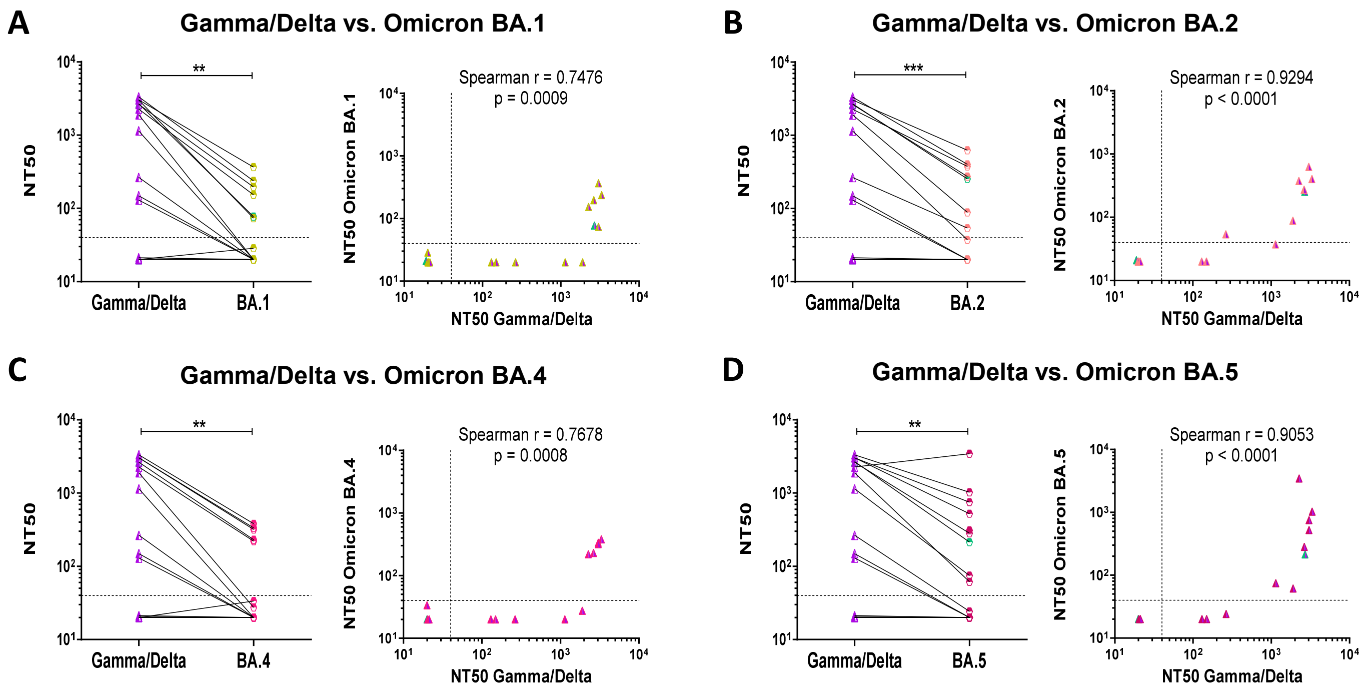

Supplementary Figure S6. Pairwise comparison and correlation of 50% neutralizing titers (NT50) of BTI sera against Delta and Omicron sublineages. NT50 between Delta and Omicron sublineages BA.1 (**A**), BA.2 (**B**), BA.4 (**C**) and BA.5 were compared (**D**). Cells were infected with the strain indicated on the x-axis. Paired samples (left panel) and Spearman correlation (right panel) are shown. The dotted line represents the 1:40 serum dilution cut-off. Sera which did not neutralize SARS-CoV-2 at the 1:40 dilution were considered non-neutralizing. Gamma-BTI are identified with green symbols. Differences between groups were compared using Wilcoxon signed rank test. The Spearman correlation coefficient (r) is indicated. P-values < 0.05 were considered significant. *: p<0.05; **: p<0.01; ***: p<0.001.
